# Female clients’ gender preferences for frontline health workers who provide maternal, newborn and child health (MNCH) services at primary health care level in Nigeria

**DOI:** 10.1186/s12913-020-05251-0

**Published:** 2020-05-19

**Authors:** Ekechi Okereke, Godwin Unumeri, Akinwumi Akinola, George Eluwa, Sylvia Adebajo

**Affiliations:** Population Council, 16 Mafemi Crescent, Off Solomon Lar Way, Utako, Abuja, Nigeria

**Keywords:** Gender preferences, Frontline health workers, Primary healthcare, Nigeria

## Abstract

**Background:**

In Nigeria, anecdotes abound that female clients, particularly within northern Nigeria, have gender-based preferences for frontline health workers (FLHWs) who provide healthcare services. This may adversely affect uptake of maternal newborn and child health services, especially at primary healthcare level in Nigeria, where a huge proportion of the Nigerian population and rural community members in particular, access healthcare services. This study explored female clients’ gender preferences for frontline health workers who provide maternal, newborn and child healthcare (MNCH) services at primary healthcare level in Nigeria.

**Methods:**

The study adopted a cross-sectional quantitative design with 256 female clients’ exit interviews from selected primary health facilities within two States - Bauchi (northern Nigeria) and Cross-River (southern Nigeria). Data was collected using Personal Digital Assistants and data analysis was done using SPSS software. Descriptive analysis was carried out using percentage frequency distribution tables. Bivariate analysis was also carried out to examine possible relationships between key characteristic variables and the gender preferences of female clients involved in the study.

**Results:**

Out of 256 women who accessed maternal, newborn and child health services within the sampled health facilities, 44.1% stated preference for female FLHWs, 2.3% preferred male FLHWs while 53.5% were indifferent about the gender of the health worker. However only 26.6% of female clients were attended to by male FLHWs. Bivariate analysis suggests a relationship between a female client’s health worker gender preference and her pregnancy status, the specific reason for which a female client visits a primary healthcare facility, a female client’s location in Nigeria as well as the gender of the health worker(s) working within the primary healthcare facility which she visits to access maternal, newborn and child health services.

**Conclusions:**

The study findings suggest that female clients at primary healthcare level in Nigeria possibly have gender preferences for the frontline health workers who provide services to them. There should be sustained advocacy and increased efforts at community engagement to promote the acceptability of healthcare services from male frontline health workers in order to have a significant impact on the uptake of MNCH services, particularly within northern Nigeria.

## Background

The health workforce is a key component of the health system [1]. The right numbers and distribution of health workers are crucial for effective health systems performance [2, 3]. But in many developing countries, prevailing health workforce shortages are further worsened by the uneven distribution of the available health workers along rural/urban divides [4]. In Nigeria, there are challenges with the numbers and distribution of frontline health workers (FLHWs) particularly nurses, midwives and community health workers as well as the access to and uptake of maternal, newborn and child health (MNCH) services at primary healthcare level [5]. These challenges appear exacerbated by assertions that there may be gender based preferences for healthcare providers at primary healthcare facilities, further complicated by gender inequity within the health workforce of many developing countries [6, 7]. These could adversely affect uptake of maternal, newborn and child health (MNCH) services particularly at primary healthcare level and within rural communities.

These challenges and concerns are against the backdrop of high maternal, newborn and child morbidity and mortality in Nigeria. It is estimated that more than 50% of all maternal deaths across the world can be linked to six focal countries including Nigeria [8] with the country’s National maternal mortality rate estimated at 576 deaths per 100,000 live births [9]. The maternal mortality rate within rural areas (828 deaths per 100,000 live-births) is estimated to be more than twice that in urban areas (351 deaths per 100,000 live-births) [10]. Among other factors, these indices appear linked to inadequate numbers of frontline health workers and health workforce distribution challenges especially within rural Nigerian communities.

Frontline health workers are critical for effective maternal, newborn and child health service delivery [11]. The effective distribution and utilization of frontline healthcare workers at primary healthcare level should be a key strategy for improving access to key MNCH services [12]. This argument is compelling especially considering the critical role of these frontline health workers in the provision of much needed healthcare services that impact heavily on maternal newborn and child health outcomes in many developing countries including Nigeria. However, it is important to generate evidence around the assertions of gender-based preferences for frontline healthcare providers. This is especially important because analysis of health systems using a gender lens and evidence arising therefrom are currently limited within the existing literature [13]. Efforts to generate such evidence and recommendations to holistically address these challenges will ultimately contribute to the global human resources for health agenda for universal health coverage amidst the post-2015 sustainable development goals (SDGs).

The human resources for health (HRH) project implemented by Population Council and the World Health Organization in Nigeria carried out a study within rural communities to investigate female clients’ gender preferences for the frontline health workers who provide maternal, newborn and child health (MNCH) services at primary health care (PHC) level using Bauchi and Cross River states as study sites. As defined within the HRH project and applied in this research study, frontline health workers here refer to nurses, midwives and community health workers i.e. community health extension workers (CHEWs), junior community health extension workers (JCHEWs) and community health officers (CHO), who tend to have the greatest access to clients and patients and provide initial care to persons in need of health services at primary healthcare level.

## Methods

### Study objectives and theory of change

As public health practitioners and policy makers strive to achieve universal health coverage and optimize the use of primary healthcare services, this study was designed to investigate if female clients have gender preferences for frontline health workers who provide services to them within primary healthcare settings. Previous studies from Nigeria and other countries have not investigated gender-based preferences of patients/clients for frontline health workers working at primary healthcare level who provide maternal, newborn and child healthcare (MNCH) services nor have previous studies investigated the implications of gender-based preferences for the uptake of MNCH services at the primary healthcare level. This study is based on a theory of change which assumes that to achieve universal health coverage and improve health outcomes within health systems, the implementation frameworks of governments and other key stakeholders should increasingly take gender issues into consideration. Gender-based considerations are important as these may have implications for the willingness to access healthcare services and the uptake of maternal, newborn and child health services by patients and clients.

### Study sites

This research study was undertaken in two States (i.e. Bauchi and Cross River States) within Nigeria. Bauchi State is in the north-eastern part of the country with a predominantly Muslim population. Cross River State is in the southern part of Nigeria and has a predominantly Christian population. For Bauchi State, the rural Local Government Areas (LGAs) selected for the research study were Alkaleri and Giade LGAs while for Cross River State, the selected LGAs were Etung and Yala LGAs.

### Study design and sampling procedure

The study was based on a cross-sectional quantitative research design. For site selection, multi-stage sampling was applied - the list of LGAs in Bauchi and Cross River States were stratified into urban and rural, which was followed by a random selection of two rural LGAs per State (Bauchi: Alkaleri and Giade LGAs; Cross River State: Yala and Etung LGAs) from the list of rural LGAs in each state. A list of PHC facilities offering maternal, newborn and child health services was stratified as Health posts, Primary Health Clinics and Primary Healthcare Centres; subsequently an equal representation of the different types of facilities was selected. Sixty-six (66) randomly selected primary healthcare facilities in Bauchi and Cross River States were involved. These health facilities represent half of all the available primary healthcare facilities in the randomly selected rural LGAs within the study States, based on a sampling approach used by Adeniyi and colleagues [14].

The researchers applied a purposive sampling strategy for client selection such that a cross-section of female clients who were accessing maternal newborn and child health services from primary healthcare health facilities were involved in this study. This study was part of a larger study undertaken to assess primary healthcare service delivery, using 66 health facilities across two states in Nigeria. For this study focused on assessing female clients’ gender preferences, the selection criteria was such that only health facilities among the 66 health facilities which recorded frontline health workers attending to an average of at least 7 patients/clients per day (determined by analysing data from the larger study) were purposively included within the study sample for this study assessing female clients’ gender preferences. Based on this selection criteria*,* all female clients who accessed the selected health facilities for maternal, newborn and child health services during the data collection period of the larger study were enrolled in this study. Thus, 256 women who accessed MNCH services from frontline healthcare providers in the selected health facilities for the study, either for themselves, their newborn or under-five children and willing to participate in the study were enrolled to participate in client exit interviews for the study.

### Data collection and analysis

Pre-tested questionnaires (see Additional file 1) were uploaded on personal digital assistants (PDAs) to facilitate the data collection process. During fieldwork the study supervisors made spot-checks on completed questionnaires, and any irregularities were corrected before the data was sent to the database designed for the study. Data analysis was carried out with SPSS software. Descriptive analysis of the data was carried out using percentage frequency distribution tables. Bivariate analyses of the data were also conducted. Fishers’ Exact test was employed during bivariate analysis (partly due to the small sample size) to explore relationships between female clients’ gender preference and some key characteristic variables such as location, pregnancy status and gender of health worker(s) seen at health facilities.

## Results

### Distribution of female clients by key characteristic variables

Two hundred and fifty-six (256) female clients were enrolled into the study. Data from the study illustrate that 58% and 42% of these female clients were from Cross River and Bauchi States respectively. About 43% of the female clients came to the health facility for maternal health-related services i.e. antenatal care, family planning and postnatal care while 55% of the female clients came for child health-related services (child nutrition, immunization, childhood illnesses), 2% came for both maternal and child health services (Table 1).

**Table 1:** Distribution of exit clients by key characteristic variables (*N* = 256)

Characteristic	(N)	Percentage (%)
State		
Bauchi	108	42.2
Cross River	148	57.8
Reason for visiting the health facility		
Myself	111	43.3
My Child	140	54.7
Myself and my child	5	2.0
Pregnancy status		
Yes	99	38.7
No	157	61.3
^a^Purpose of visit for Self		
ANC	96	37.5
Childbirth Care (neonate)	4	1.6
Delivery	3	1.2
Post-Partum/Post–Natal care	19	7.4
Family Planning	5	2.0
^a^Purpose of visit for the Child		
Childbirth care (under 5 yrs)	3	1.2
Child Nutrition	1	0.4
Child Immunization	78	30.5
Childhood illness	52	20.3
Cadre of health care provider seen		
JCHEWs	39	15.2
CHEWs	114	44.5
CHOs	26	10.2
Nurses	50	19.5
Midwives	5	2.0
Don’t know	22	8.6
Gender of health care provider seen		
Male	68	26.6
Female	188	73.4

^a^Total frequency (N) i.e. ‘Purpose of visit for Self’ + ‘Purpose of visit for the Child’ was more than 256 due to multiple responses. Five female clients were attending the health facility for healthcare services for both themselves and their child

### Female clients’ gender preferences versus attendance of FLHWs’ to female clients

Tables 2, 3, 4 and 5 show that out of 256 women who visited the health facilities, 44.2% indicated preference for female frontline health workers, 2.3% preferred male frontline while 53.5% were indifferent about the gender of the health worker. However only 27% of female clients were attended to by male FLHWs. By States, less than 1% of the female clients prefer male frontline health workers, almost 60% prefer female frontline health workers and around 40 % (39.8%) have no health worker gender preference in Bauchi State. For Cross River State, 3% of female clients preferred male frontline health workers, about one-third of the clients interviewed preferred female clients while over 60% stated that they had no frontline health worker gender preference. In Bauchi State, about two-thirds of the clients interviewed were attended to by a female health worker while in Cross River state, almost 80% of clients were attended to by female health workers.

**Table 2:** Clients’ frontline health worker gender preference (*N* = 256)

	N	%
Clients frontline health worker gender preference		
Client preference for Male	6	2.3
Client preference for Female	113	44.2
No gender preference	137	53.5
Total	**256**	**100%**

**Table 3:** Clients’ frontline health worker gender preference by states (*N* = 256)

	Bauchi		Cross River	
	N	%	N	%
Client preference for Male	1	0.9	5	3.4
Client preference for Female	64	59.3	49	33.1
No gender preference	43	39.8	94	63.5
Total	**108**	**100%**	**148**	**100%**

**Table 4:** Frontline health workers’ attendance to clients (*N* = 256)

	N	%
Frontline health workers’ attendance to clients		
Client attended by male FLHW	68	27
Client attended by a female FLHW	188	73
Total	**256**	**100%**

**Table 5:** Frontline health workers’ attendance to clients by states (*N* = 256)

	Bauchi		Cross River	
	N	%	N	%
Client attended by male FLHW	37	34.3	31	20.9
Client attended by a female FLHW	71	65.7	117	79.1
Total	**108**	**100%**	**148**	**100%**

### Bivariate analysis

Fisher’s exact test was used during bivariate analysis and this was deemed most appropriate, based on the small number of responses across categorical variables. Table 6 presents the summary results of bivariate analysis between key characteristic variables and gender preferences of female clients from the study. The analysis suggests a relationship between location i.e. either Bauchi or Cross River State and female clients’ health worker gender preference (*p* < 0.001). Similarly, bivariate analysis suggests a relationship between clients’ pregnancy status and their health worker gender preference (*p* < 0.001) as well as a relationship between female clients’ health workers gender preference and the reason for visiting the primary healthcare facility either for the client’s self, her child or for both herself and her child (*p* < 0.001). The results also suggest a relationship between the gender of health worker(s) working within the primary healthcare facility and female clients’ health workers gender preference (*p* < 0.001).

**Table 6:** Bivariate analysis of key characteristic variables against client’s FLHW gender preference

Key characteristic variables	Male health care provider	Female health care provider	Doesn’t Matter	
State				*P*-value
Bauchi	0.9%	59.3%	39.8%	.000
Cross River	3.4%	33.1%	63.5%	.000
Reason for visiting the health facility				
Myself	0.0%	58.6%	41.4%	
My Child	4.3%	32.1%	63.6%	
Myself and my child	0.0%	60.0%	40.0%	
Pregnancy status				.000
Yes	0.0%	59.6%	40.4%	
No	3.8%	34.4%	61.8%	
Purpose of visit for MNCH services				.124
ANC	0.0%	60.4%	39.6%	
Childbirth Care	0.0%	75.0%	25.0%	
Delivery	0.0%	0.0%	100.0%	
Post-Partum/Post –Natal care	0.0%	37.5%	62.5%	
Family Planning	0.0%	80.0%	20.0%	
Cadre of healthcare worker seen				.105
JCHEWs	0.0%	38.5%	61.5%	
CHEWs	2.6%	49.1%	48.2%	
CHOs	3.8%	30.8%	65.4%	
Nurses	4.0%	32.0%	64.0%	
Midwives	0.0%	40.0%	60.0%	
Don’t know	0.0%	72.7%	27.3%	
Gender of healthcare worker seen				
Male	4.4%	23.5%	72.1%	0.000
Female	1.6%	51.6%	46.8%	

## Discussion

This research study was designed to investigate whether female clients have gender preferences for frontline health workers who provide maternal, newborn and child health (MNCH) services at primary healthcare level in Nigeria. Currently, there are few studies that have sought to investigate female clients’ health worker gender preference and to understand the implications for access to as well as uptake of health services at primary healthcare level [15]. In a British study by Nicholas, less than 10% of female respondents stated preference to consult female primary healthcare workers for general health problems but more than half of the respondents stated preference for female primary health workers for maternal health problems [16]. The finding from this study (Tables 2) that there is relatively low stated preference to access maternal, newborn and child health services from male frontline health workers by female clients within primary healthcare facilities raises serious concerns and it also reinforces the perception that gender-based preferences exist for services from health workers at primary healthcare level. The preference of female clients to receive MNCH services from female frontline healthcare workers was reportedly higher in Bauchi (northern Nigeria) compared to Cross-River (southern Nigeria) (Table 3). In addition, the finding that more than half of the clients interviewed indicated that they have no health worker gender preference is also an important finding. This indifference to the gender of health workers providing services at primary healthcare level was much higher in Cross River State (southern Nigeria) than in Bauchi State (northern Nigeria) (Table 3). In gender conservative contexts (such as within northern Nigeria), there is greater preference for female health workers providing MNCH services to female clients [17], which is supported by the findings from this research study. Furthermore, it is noteworthy that female health workers in both States actually provided more MNCH services to female clients (Table 5), reiterating a clear preference among female clients to receive healthcare services from female frontline health workers. This corroborates research findings reported elsewhere that female clients tend to prefer accessing MNCH services from female health workers, especially in primary healthcare settings [15]. This will invariably have an impact on the uptake of crucial MNCH services as well as health outcomes at primary healthcare level in Nigeria. This preference of female clients for female health workers may be associated with suggestions by some social scientists and the perception that maternal healthcare services are more likely to be better understood and delivered by female healthcare providers [18].

Bivariate analyses from this study suggest a relationship between a female client’s health worker gender preference and the following: her pregnancy status, the specific reason for which female clients visit primary healthcare facilities to access maternal, newborn and child health services, the gender of the health worker(s) seen/working within the primary healthcare facilities she accesses and the female client’s location in Nigeria (Table 6). In other words, where a female client is located within Nigeria, whether she is pregnant or not, the gender of the health worker(s) working within the health facility as well as the reason for which she seeks MNCH services at health facilities are key factors likely to affect the gender of the health worker she will prefer to receive health services from, particularly within primary healthcare settings. These have serious implications for the uptake of MNCH services at primary healthcare level in Nigeria.

These study results and their implications emphasize the need for gender related issues to be given more serious consideration during the production, hiring and deployment processes for frontline healthcare workers [13] as well as during efforts towards health system strengthening more broadly [17]. Gender mainstreaming should be incorporated more strategically within the planning processes as well as implementation frameworks for human resources for health (HRH) management [13] especially at the primary healthcare level in Nigeria. In addition, there should be increased efforts at advocacy and community engagement to promote the acceptability of maternal, newborn and child health services from male frontline health workers, especially within northern Nigeria, to significantly improve uptake of MNCH services and consequently reduce maternal, newborn and child morbidity and mortality, particularly in rural communities across Nigeria.

The study has some key limitations: first the findings from this study apply to specific cadres of frontline health workers i.e. nurses, midwives and community health workers. Second, the study was conducted in rural communities and at primary healthcare level and as a result, it does not provide insights into female clients’ gender preference for frontline health workers in urban settings as well as within other levels of care i.e. secondary or tertiary levels of healthcare in Nigeria. Another limitation is that the sample size of the study is relatively small i.e. 256 female clients across two study states, however this is the number of female clients who consented to participate in the study via client exit interviews. Lastly, the study was conducted in only two out of the 36 States in Nigeria and thus cannot be generalized to the whole of Nigeria, however both States represent on large scale two major classifications within Nigeria: a conservative and predominantly Muslim group in Bauchi and a liberal and predominantly Christian group in Cross River. Thus, the results broadly capture female clients’ preferences for the gender of healthcare providers at primary healthcare level in Nigeria.

## Conclusion

In conclusion, female clients appear to have gender preferences for the frontline health workers providing maternal newborn and child health services at primary healthcare level in Nigeria. The study findings demonstrate that there may be challenges with the acceptability of maternal, newborn and child health services from male frontline health workers by female clients, especially within northern Nigeria. This suggests that cultural norms, stereotypes and practices still have considerable influence on the uptake of MNCH services in Nigeria. These issues need to be addressed to increase the uptake of MNCH services at primary healthcare level in Nigeria.

## Supplementary information

**Additional file 1.** Exit interview questionnaire for female clients

## Data Availability

The datasets for this study are available from the corresponding author upon reasonable request. The female client exit interview questionnaire is included as a supplementary file.

## Abbreviations

ANC: Antenatal care
CHEW: Community health extension workers
CHO: Community health officer
FLHWs: Frontline health workers
GAC: Global Affairs Canada
HRH: Human Resources for Health
IRB: Institutional review board
JCHEW: Junior community health extension worker
LGA: Local Government Area
MNCH: Maternal newborn and child health
PDA: Personal digital assistant
PHC: Primary health care
SDG: Sustainable development goals
SPSS: Statistical package for social sciences
WHO: World Health Organization
