# Supplementary material for: Female clients’ gender preferences for frontline health workers who provide maternal, newborn and child health (MNCH) services at primary health care level in Nigeria
Source: BMC Health Serv Res. 2020 May 19;20:441. doi: 10.1186/s12913-020-05251-0 (PMC7236331; doi:10.1186/s12913-020-05251-0)
Supplement: Supplementary file 1 — Additional file 1. Exit interview questionnaire for female clients [file 12913_2020_5251_MOESM1_ESM.pdf]

Exit Interview Questionnaire for Female Clients **Date ______________________________**

General guidelines to interviewers:

1. Identify women of reproductive age (15 -49 years) who are leaving the health facility.
2. Introduce yourself and ask if you can ask a few questions which will not take more than 5 minutes. Explain to each woman that the questions are not personal and are anonymous but will help in improving services provided in health facilities.
3. If the female client/patient agrees to be interviewed, start the interview with the following preamble:

You are being invited to take part in a research study. This study will involve interviewing women who are visiting primary healthcare facilities to access healthcare services from health workers. This is part of a larger study which aims to better understand primary healthcare service delivery in this State and then make recommendations about how to better improve healthcare services for patients/clients. The interview for this study will last for not more than 5 minutes.

Before you decide whether to participate, you may need to understand why the research is being done and what it would involve. You may talk to others about the study if you wish. Please ask me if there is anything that is not clear. If you feel that you understand the purpose of this study or when all of your questions have been answered (if you have any questions), you will be asked if you wish to participate in the study, and if yes, to sign an 'Informed Consent Form'. You will be given a signed copy to keep.

Do you agree to participate in this interview for the study?

Yes ___________

No ___________

Instructions:

a. Consent Instructions for Interviewer: Give a copy of the 'Informed Consent Form' to the female client or read the standard consent text from the Informed Consent Form to the female client if she agrees to participate in the exit interview. Each female client who agrees to participate must sign the informed consent form.
b. Interviewer to fill/complete the exit interview questionnaire.

QUESTIONS FOR FEMALE CLIENTS

1) **State** ______________________________

2) Are you currently pregnant?

Yes _________

No _________

3) For whom did you come to this primary health care facility today?

Myself Yes ______________ No ___________________ (If Yes, go to 4a)

My child Yes ______________ No ___________________ (If Yes, go to 4b)

**If female client indicates that she came for both herself and her child, then complete 4a & 4b*

4a) **Why did you come to see the health worker(s) today?**

Antenatal Care Yes ______________ No ___________________

Delivery Yes ______________ No ___________________

Childbirth Care (Neonate) Yes ______________ No ___________________

Postpartum/Post-Natal Care Yes ______________ No ___________________

Family Planning Yes ______________ No ___________________

Others (Please specify) ____

4b) **Why did you come to see the health worker(s) today?**

Childbirth Care (Under five years) Yes ____________ No ___________________

Child Nutrition Yes ____________ No ___________________

Child Immunization Yes ____________ No ___________________

Childhood Illness Yes ____________ No ____________________

5) Who attended to you today?

JCHEW Yes ____________ No ___________________

CHEW Yes ____________ No ___________________

CHO Yes ____________ No ___________________

Nurse Yes ____________ No ___________________

Midwife Yes ____________ No ___________________

6) **Is the health worker who attended to you male or female?**

Male ___________

Female ___________

7) **Will you prefer to receive services from a male or female health worker at the health facility?**

Male ___________

Female ___________

I do not care about the gender of health worker ____________

8) **Are you satisfied with the services you received from the health worker who attended to you?**

Yes ____________ No _______________

Thank you for your participation in this interview/study.
